# Burden of influenza-associated respiratory and circulatory mortality in India, 2010-2013

**DOI:** 10.7189/jogh.10.010402

**Published:** 2020-06

**Authors:** Venkatesh Vinayak Narayan, A Danielle Iuliano, Katherine Roguski, Rohit Bhardwaj, Mandeep Chadha, Siddhartha Saha, Partha Haldar, Rajeev Kumar, Vishnubhatla Sreenivas, Shashi Kant, Joseph Bresee, Seema Jain, Anand Krishnan

**Affiliations:** 1 Centre for Community Medicine, All India Institute of Medical Sciences, New Delhi; 2 Influenza Division, Centers for Disease Control and Prevention, Atlanta, Georgia, USA; 3 SRS division, Office of Registrar General of India, New Delhi, India; 4 National Institute of Virology, Pune, India; 5 Influenza Division, Centers for Disease Control and Prevention, New Delhi, India; 6 Department of Biostatistics, All India Institute of Medical Sciences, New Delhi

## Abstract

**Background:**

Influenza causes substantial morbidity and mortality worldwide, however, reliable burden estimates from developing countries are limited, including India. We aimed to quantify influenza-associated mortality for India utilizing 2010-2013 nationally representative data sources for influenza virus circulation and deaths.

**Methods:**

Virological data were obtained from the influenza surveillance network of 10 laboratories led by National Institute of Virology, Pune covering eight states from 2010-2013. Death data were obtained from the nationally representative Sample Registration System for the same time period. Generalized linear regression with negative binomial distribution was used to model weekly respiratory and circulatory deaths by age group and proportion of specimens positive for influenza by subtype; excess deaths above the seasonal baseline were taken as an estimate of influenza-associated mortality counts and rates. Annual excess death rates and the 2011 India Census data were used to estimate national influenza-associated deaths.

**Results:**

Estimated annual influenza-associated respiratory mortality rates were highest for those ≥65 years (51.1, 95% confidence interval (CI) = 9.2-93.0 deaths/100 000 population) followed by those <5 years (9.8, 95% CI = 0-21.8/100 000). Influenza-associated circulatory death rates were also higher among those ≥65 years (71.8, 95% CI = 7.9-135.8/100 000) as compared to those aged <65 years (1.9, 95% CI = 0-4.6/100 000). Across all age groups, a mean of 127 092 (95% CI = 64 046-190,139) annual influenza-associated respiratory and circulatory deaths may occur in India.

**Conclusions:**

Estimated influenza-associated mortality in India was high among children <5 years and adults ≥65 years. These estimates may inform strategies for influenza prevention and control in India, such as possible vaccine introduction.

Worldwide, between 291 000 and 646 000 people die each year from seasonal influenza-associated respiratory illnesses [1], 36% of which occur in low and middle income countries (LMICs) like India. In India, a tropical climate and lower middle income country with a population of approximately 1.2 billion people (17% of world population), influenza virus circulation usually peaks during monsoon season (June-September) with secondary peaks during winter periods (November-February) [2]. Furthermore, the actual timing of the influenza season varies across the country due to different climatic regions [2]. Previous studies have estimated the morbidity burden of severe acute lower respiratory tract infection and hospitalization rates for children in India due to influenza [3,4]. However, there are no national estimates of influenza-associated deaths available for India.

Estimating influenza mortality is complicated by the fact that influenza is often not listed as underlying cause on death certificates, thus influenza-associated mortality estimation is often conducted using indirect estimation methods with statistical modeling [5,6]. These indirect methods rely on national vital records systems as well as consistent surveillance data, which has also hampered many estimates in LMICs such as India due to issues with the quality or availability of necessary data sources [6-8]. However, estimates from the lower resource and tropical climate settings can be important to understand the true burden of influenza on high risk age groups and populations. For example, an earlier study aimed at estimating the global burden of influenza by utilizing data from systematic review of studies found that 99% of influenza deaths in children <5 years occurred in developing countries [9]. Several studies have explored the burden of influenza-associated deaths for middle-income countries, including countries in the Americas [10], as well as Asian countries, such as China, Thailand, and Bangladesh [11-13].

Influenza virus infection incidence is known to vary by season and vaccination campaigns in many countries occur at the beginning of the influenza season to decrease potential morbidity and mortality [14]. However, influenza vaccination is currently a low priority vaccine in India. Currently, the Ministry of Health and Family Welfare (MoHFW), Government of India recommends annual influenza vaccination for high risk groups, specifically, health care workers, pregnant women, and people with chronic health conditions. For children <5 years and adults ≥65 years, the seasonal influenza vaccine has been categorized as ‘desirable’ or lower priority [15]. While MoHFW recommends seasonal influenza vaccine, they do not routinely purchase or provide the vaccine for these age groups. Estimates of influenza mortality for India could guide policy for prevention and control measures, such as the introduction of seasonal influenza vaccine in public health programmes by identifying the burden among populations at risk of influenza-associated death. In this context, we aimed to estimate influenza-associated mortality for India using national level vital records and viral surveillance data.

## METHODS

### Mortality data: Sample Registration System (SRS)

We obtained weekly death data for 2010 through 2013 collected through the Sample Registration System (SRS) from the Office of Registrar General of India. The four years provided by the SRS were the most recent nationally representative mortality data available for India at the time of our request due to time required to clean and systematically code deaths. The SRS is a routine annual demographic survey, serving as the primary system for collecting data on births and deaths in India since 1971. Details of the SRS, including design, sampling scheme, physician assignment of the underlying cause of death and the methodology have been described elsewhere [8,16,17]. In brief, the SRS divided each state or union territory of India into one million sample units based on the population using the 2001 census. SRS selected 7597 sampling units (4433 rural and 3164 urban units) by stratified simple random sampling across all states of India, with a population of approximately 7.6 million (0.6%) for continuous monitoring of births and deaths [18]. These sample units are representative of the population at the state level and are the only source of nationally representative mortality data in India.

Within the SRS system, trained surveyors record deaths. Verbal autopsy (VA) methods, which include interviewing surviving household members, are used to capture cause of death. SRS teams visit communities that are part of this system throughout the year to identify deaths and collect information on possible causes of death. VA reports are sent through a web-based platform to two trained physicians who assign the most probable underlying cause of death based on guidelines from International Classiﬁcation of Diseases (ICD) coding system [8,16,17]. For our analysis, deaths were categorized into groups based on codes from the tenth revision of ICD (ICD-10): circulatory diseases (ICD-10 codes I00-I99) and respiratory diseases (ICD-10 codes J00-J99). We obtained weekly counts of respiratory and circulatory deaths for 2010-2013 by three age groups (<5, 5-64, ≥65 years). To calculate rates, we also obtained age group-specific annual population estimates for the SRS sampling area for 2010-2013 from the SRS annual reports [18].

### Viral surveillance data

The Indian Council of Medical Research (ICMR) established a laboratory-based surveillance network for influenza virus, which is coordinated by the National Institute of Virology (NIV), Pune, India [2]. The virology laboratories were located across ten cities in eight states providing geographic and climatic representation of India. Patients with influenza-like illness (ILI) presenting at outpatient departments and hospitalized patients with severe acute respiratory infection (SARI) were randomly selected and enrolled into the surveillance system, which has been described elsewhere [2]. ILI was defined as sudden onset of fever >38°C or history of fever in the past three days with cough, sore throat or rhinorrhea [7]. SARI was defined as an ILI episode with difficulty breathing or clinically suspected pneumonia (in children <5 years) with an increased respiratory rate requiring hospitalization, following the Integrated Management of Childhood Illness definitions [19].

Specimens were tested for influenza viruses using reverse-transcription polymerase chain reaction (RT-PCR) following standardized protocols coordinated by NIV, which is also the World Health Organization (WHO) designated National Influenza Center for India. Surveillance nurses collected five to ten nasopharyngeal swabs each week from participating centers from 2010 to 2013 year-round [2]. These data are collected systematically throughout the year to demonstrate the timing of inﬂuenza virus circulation throughout India. We obtained weekly numbers of specimens positive for inﬂuenza viruses by type (A or B) and influenza A subtype (H1N1pdm09 or H3N2) and the total number of specimens tested. Due to limited viral surveillance data for the age groups in this analysis, viral surveillance data was combined by age group and an all-age percent positive was used in models. The weekly influenza percent positive was calculated combining data for all ages and by dividing the total number of specimens that tested positive for influenza viruses divided by the total number of specimens collected in that week.

### Evaluation of influenza circulation and mortality data

Before conducting this analysis, these data underwent a detailed evaluation described elsewhere using specific criteria for robustness of data, including sample size, proportion of ill-defined deaths, and representativeness to determine quality of data and guide methodology to estimate influenza-associated mortality [8].

**Figure 1:**
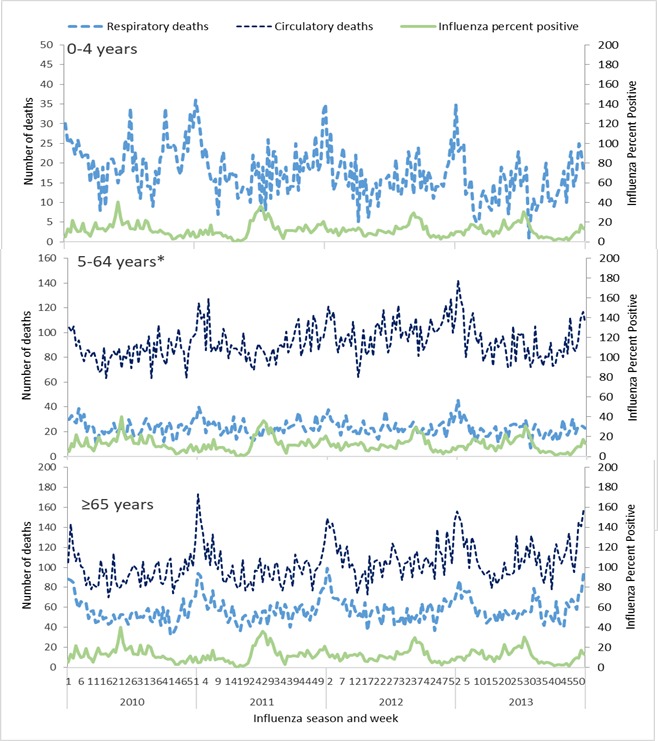
Sample registration system respiratory and circulatory coded deaths by week and influenza virus percent positive by week, India 2010-2013. *Circulatory deaths were for age group <65 years due to very small number of deaths in age group 0-4 years (includes under 5 circulatory deaths).

Although the influenza percent positive may vary between age groups, we utilized an overall influenza percent positive combining surveillance information for all age groups. This approach was selected because of potential biases in the surveillance data including who is enrolled in surveillance, health care seeking behaviours in the population, and limited numbers of specimens collected and tested for influenza by age group. By age group for the period of study, we graphed the weekly influenza percent positive along with weekly count of deaths coded as respiratory or circulatory (**Figure 1**).

### Estimating influenza-associated deaths:

We used generalized linear regression with a negative binomial distribution, stratified by three age groups (<5, 5-64, and ≥65 years), to model weekly counts of respiratory and circulatory deaths and incorporating the influenza percent positive for all ages to attribute deaths to influenza. The model was constructed with weekly respiratory or circulatory death counts as the dependent variable and incorporated influenza virus subtypes as independent variables. We assumed a negative binomial distribution of respiratory and circulatory deaths to account for over-dispersion in the count data. A continuous time term counting the available weeks of death data was utilized to adjust for trends over time, and harmonic terms were used to adjust for the assumed sinusoidal and seasonal pattern of deaths over time. Both time and harmonic terms were used to estimate the baseline number of respiratory or circulatory deaths. We used a log link function in the models, which assumes a multiplicative relationship between the exposure to inﬂuenza virus infection and resulting mortality. The models included independent variables that comprised of the weekly percentage of specimens testing positive for influenza A(H3N2), influenza A(H1N1)pdm09, and inﬂuenza B viruses during a given week. A population offset was used to account for annual changes in population for each age group. Weekly estimates were summarized by calendar year and subsequently used to calculate national estimates of influenza-associated deaths for India.

We evaluated different models considering inclusion of alternative terms for patterns of influenza virus activity using viral surveillance data to identify the best model for estimating influenza-associated deaths (Table S1 in **Online Supplementary Document**). We evaluated model fit using the Akaike Information Criterion (AIC) value and statistical significance of viral terms. We explored an additive model using a linear link to confirm our initial assumption that the relationship between influenza and death was multiplicative. To evaluate baseline death estimates for respiratory and circulatory deaths, we compared the use of harmonic terms (sine and cosine) to the use of splines. Additionally, since deaths may not align with actual timing of viral infection, we examined the use of different lag periods for the viral data by plotting death data in conjunction with viral surveillance data for visual assessment. In our models, we explored using viral terms with no lag in time period, a one-week lag or a two-week lag to evaluate the best model fit and to evaluate which viral terms were associated with the selected death outcome.

In our evaluation of viral surveillance data, we observed that during some of the weeks only a few specimens were tested, which may have caused the percent positive to be unstable between weeks. Thus, we also examined a different approach to calculate the percent positive referred to as the “standardized percent positive” for influenza viral terms. The standardized percent positive was calculated by dividing the weekly number of specimens positive for influenza by the total of the number of specimens tested during the calendar year.

A detailed description of the models explored is provided in the Appendix (Table S1 in the **Online Supplementary Document**). The final model selected included sine and cosine terms to estimate the baseline, one time term, and the influenza virus subtypes as indicator variables:

In each regression model,

Y_i_ = αexp {β_0_ + β_1_[t_i_] + β_2_[sin(2t_i_π/52.179)] + β_3_[cos(2t_i_π/52.179)] + β_4_[A(H3N2)_i_] + β_5_[A(H1N1pdm09)_i_] + β_6_[B_i_]}

where: Y_i_ was the number of deaths in a particular week i, α is log of population (offset variable), t_i_ was time in weeks and A(H3N2)_i_, A(H1N1pdm09)_i,_ and B_i_ were the percentages of specimens testing positive for each influenza virus subtype respectively in a given week, without a lag.

Stratified models were run for each death category (respiratory and circulatory) and age group (<5, 5-64, and ≥65 years). The mortality associated with a specific influenza type or subtype was calculated by first obtaining the predicted weekly deaths from the model that included a term for the specific influenza type or subtype (full model). This estimate was then subtracted from the predicted deaths obtained from the model wherein the terms for the for the specific influenza type or subtype was set to zero (baseline model). Only positive differences obtained after subtracting the weekly deaths from full model to predicted weekly deaths from the baseline model were included. The same model was used for each age group and for both respiratory and circulatory deaths. All three age group models were run for respiratory deaths. As there were very few circulatory deaths in the <5 age group, it was combined with the 5-64 age group for the circulatory models, since it was not possible to run the model with such a small sample size. Thus, only two models (<65 and ≥65 years) were utilized for circulatory deaths. To account for the temporal autocorrelation of the residuals, bootstrapping was used to calculate individual week and year standard errors around these calculated estimates. The bootstrap standard errors were used to calculate corrected confidence intervals around estimates of influenza-associated mortality [20]. Statistical analyses were carried out using R version 3.3.1 (R Foundation for Statistical Computing, Vienna, Austria).

### Extrapolation to calculate a national estimate

We first used the regression models to calculate the age group-specific influenza-associated respiratory and circulatory death counts and associated standard errors by calendar year. From these deaths, rates were calculated using the age-specific SRS population denominators. The national estimate was then calculated by applying the age-specific influenza-associated respiratory and circulatory mortality rates and standard errors to the age-specific national population based on 2011 Indian national census data and adjusted for growth and death rates each year [21]. This generated a national estimate of deaths along with its standard error for each year and age group. We calculated 95% confidence intervals for annual estimates by age group. Age-specific national estimates were calculated using the year-specific death count estimates, summed across all age groups and dividing by the number of available years to obtain a mean national estimate of influenza-associated death.

## RESULTS

### Mortality data summary

From 2010-2013, approximately 45 000 (range 44 128-45 573) were reported each year through SRS (Table S2 in the **Online Supplementary Document**). Of these, a mean of 11.4% were respiratory deaths and 22.8% were circulatory deaths. On average, 58.6% of respiratory deaths for the four years were among adults ≥65 years. For circulatory deaths, an average of 52.9% of deaths were among adults ≥65 years. The overall proportion of ill-defined causes of death in the SRS data system was 12.4%. While SRS report did not provide proportion of ill-defined causes of death by <65 and ≥65 age groups, this proportion was reported to be less than 6% in population aged <70 years whereas for older adults aged ≥70 years, the proportion rose to 29%.

### Viral surveillance data summary

National viral surveillance data for 2010-2013 included 37 816 nasopharyngeal specimens tested for influenza viruses (Table S3 in **Online Supplementary Document**). Of these, 4771 (13%) specimens were positive for influenza viruses ranging from a low of 12% in year 2010 to a high of 15% in year 2011. The predominant circulating viruses in 2010 were influenza A(H1N1pdm09) with 48% and influenza B with 46% of the positive specimens, respectively. For 2011 and 2013, the predominant circulating virus was influenza A(H3N2) comprising of 56% and 61% of the positive specimens for the respective years. For 2012, influenza B was the predominant circulating virus, comprising of 59% of the positive specimens.

### Seasonality of respiratory and circulatory deaths and influenza circulation

We observed a peak in the influenza percent positive from May to September accompanied by secondary peaks throughout the year (**Figure 1**). Peak respiratory and circulatory deaths occurred during winter months (December-January), which was outside of the typical influenza season.

### Estimates of influenza-associated respiratory and circulatory deaths

**Figure 2:**
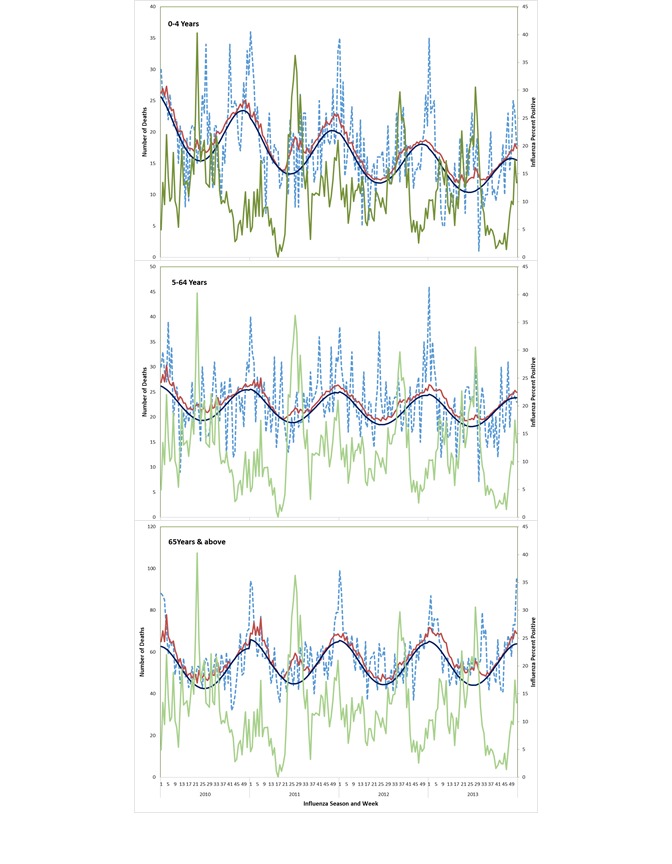
Observed respiratory deaths and predicted inﬂuenza-associated respiratory deaths and proportion positive for influenza viruses by age group, week, and year. The dotted blue line represents respiratory deaths, the green line represents influenza percent positive, the dark blue line represents expected baseline mortality, and the red line represents predicted mortality. The number of excess influenza-associated deaths are the difference between the orange and red lines

**Figure 3:**
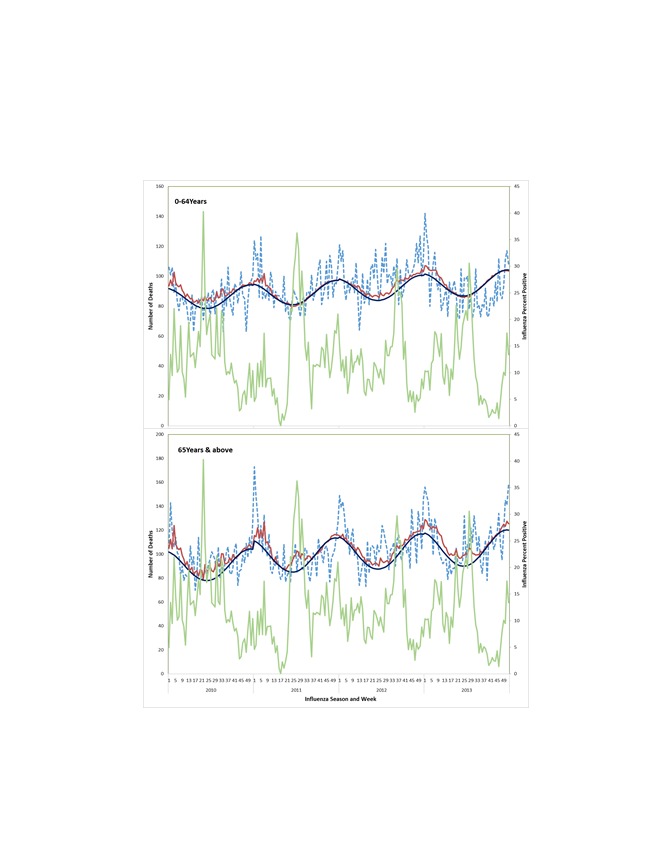
Observed circulatory deaths and predicted inﬂuenza-associated circulatory deaths and proportion positive for influenza viruses by age group, week, and year. The dotted blue line represents circulatory deaths, the green line represents influenza percent positive, the dark blue line represents expected baseline mortality, and the red line represents predicted mortality. Again, the influenza-associated excess circulatory deaths were the difference between the predicted circulatory mortality (ie, the orange line) and the baseline circulatory mortality (ie, the red line).

The peaks in inﬂuenza-associated mortality are depicted in **Figure 2** and **Figure 3** and their timings vary from year to year. Most influenza-associated deaths occur within the typical influenza season in India, which generally occurs from April to September each year. Respiratory excess death estimates were highest for those ≥65 years (51.1, 95% confidence interval (CI) = 9.2-93.0 deaths /100 000 population) followed by those <5 years (9.8, 95% CI = 0-21.8/100 000), whereas the estimates for those 5-64 years were lowest (1.1, 95% CI = 0-2.4/100 000). For children <5 years, the influenza virus subtype associated with the highest mortality rate varied by year, with the highest mortality associated with A(H1N1pdm09) in 2010, A(H3N2) in 2011 and 2013, and B in 2012, which was consistent with circulating virus subtypes during those years (**Table 1**). Among persons ≥65 years, highest rates of influenza-associated respiratory mortality were observed for influenza A viruses with limited mortality associated with influenza B viruses.

**Table 1:** Estimated influenza-associated respiratory death rate per 100 000 population by influenza subtype, year, and age group, India, 2010-2013

Age group	Influenza total (95% CI)	Influenza A(H3N2) (95% CI)	Influenza A(H1N1pdm09) (95% CI)	Influenza B (95% CI)
**<5 years**				
2010	10.5 (0-27.4)	1.1 (0-2.2)	5.8 (0-18.9)	4.8 (0-15.5)
2011	12.5 (1.1-25.8)	8.1 (0-17.2)	2.0 (0-6.3)	3.1 (0-9.9)
2012	7.4 (0.0-19.1)	1.5 (0-3.1)	2.8 (0-8.9)	4.1 (0-13.2)
2013	8.7 (0.4-17.9)	6.4 (0-13.2)	1.9 (0-6.0)	1.0 (0-3.3)
Average	9.8 (0-21.8)	4.3 (0-10.0)	3.1 (0-9.6)	3.2 (0-10.0)
**5-64 years**				
2010	1.4 (0-3.0)	0.1 (0-0.2)	1 (0-2.3)	0.4 (0-1.4)
2011	1.0 (0.0-2.3)	0.5 (0-1.3)	0.4 (0-0.8)	0.3 (0-0.9)
2012	1.0 (0-2.3)	0.1 (0-0.3)	0.6 (0-1.3)	0.4 (0-1.4)
2013	0.9 (0.0-2.0)	0.5 (0-1.3)	0.4 (0-0.9)	0.1 (0-0.4)
Average	1.1 (0-2.4)	0.3 (0-0.8)	0.6 (0-1.4)	0.3 (0-1.0)
**≥65 years**				
2010	56.4 (7.4-108.4)	4.3 (0.9-7.6)	55.4 (13.9-95.5)	5.1 (0-30.9)
2011	55.4 (19.1-96.9)	35.4 (7.7-63.8)	20.9 (5.3-36.0)	3.4 (0-20.8)
2012	35.1 (2.2-78.2)	7.1 (1.6-12.7)	31.6 (8.0-54.4)	5.1 (0-31.5)
2013	57.7 (19.5-95.0)	35.9 (8.1-63.7)	23.7 (5.9-41.3)	1.5 (0-8.9)
Average	51.1 (9.2-93.0)	20.8 (0.8-40.8)	32.7 (7.0-58.4)	3.8 (0-17.7)

CI – confidence interval

Influenza-associated circulatory death rates were higher among those ≥65 years (71.8, 95% CI = 7.9-135.8/100 000) as compared to those aged <65 years (1.9, 95% CI = 0-4.6/100 000). For both age groups, estimates across the years consistently showed more deaths associated with influenza A viruses, notably the influenza A(H1N1pdm09) virus (**Table 2**).

**Table 2:** Estimated influenza-associated circulatory death rate per 100 000 population by influenza subtype, year, and age group, India, 2010-2013

Age group	Influenza, total (95% CI)	Influenza, A(H3N2) (95% CI)	Influenza, A(H1N1pdm09) (95% CI)	Influenza, B (95% CI)
**<65 years**				
2010	2.9 (0-6.7)	0.0 (0-0.2)	2.6 (0-5.5)	0.7 (0-2.8)
2011	1.5 (0.0-4.0)	0.3 (0-1.8)	0.9 (0-2.0)	0.5 (0-1.9)
2012	2.1 (0-5.3)	0.1 (0-0.3)	1.6 (0-3.5)	0.7 (0-3.1)
2013	1.4 (0-3.9)	0.3 (0-2.0)	1.1 (0-2.4)	0.2 (0-0.8)
Average	1.9 (0 - 4.6)	0.2 (0-0.9)	1.6 (0-3.4)	0.5 (0-1.9)
**≥65 years**				
2010	85.8 (13.5-163.2)	4.5 (0-9.5)	90.7 (27.8-151.4)	5.6 (0-40.2)
2011	69.9 (19.1-132.3)	39.0 (0-83.4)	33.5 (10.3-56.0)	3.8 (0-27.2)
2012	53.3 (3.3-121.6)	7.5 (0-15.9)	56.7 (17.3-94.6)	6.1 (0-43.5)
2013	78.7 (19.2-141.0)	42.1 (0-88.3)	40.9 (12.3-69.0)	1.7 (0-12.2)
Average	71.8 (7.9-135.8)	23.4 (0-55.0)	55.2 (15.0-95.5)	4.3 (0-22.8)

CI – confidence interval

### National extrapolation

Across the years 2010-13, the highest number of respiratory and circulatory deaths occurred during 2010 (**Table 3**). The national annual age-specific influenza-associated respiratory deaths was 11 203 (95% CI = 0-24 998) among children <5 years, 11 025 (95% CI = 0-24 500) among persons 5-64 years, and 34 275 (95% CI = 6178-62 371) among adults ≥65 years. The annual total age-specific influenza-associated circulatory deaths were 22 395 (95% CI = 0-53 576) among persons <65 years and 48 194 (95% CI = 5283-91 105) among adults ≥65 years. We estimated that a total of 127 092 (95% CI = 64 046-190 139) influenza-associated respiratory and circulatory deaths across all age groups may occur annually in India.

**Table 3:** Estimated influenza-associated annual respiratory and circulatory deaths by year, and age group, India, 2010-2013

Year	Respiratory deaths (95% CI)			Circulatory deaths (95% CI)		Respiratory & Circulatory deaths (95% CI)
**<5 years**	**5 to 64 years**	**≥65 years**	**<65 years**	**≥65 years**	**All age**
**2010**	11 776 (0-28 928)	13 818 (0-30 164)	36 996 (4038-69 955)	33 011 (0-72 776)	56 314 (7494-105 135)	151 915 (76 999-226 830)
**2011**	14 262 (0-28 664)	10 626 (0-23 129)	36 881 (10 883-62 880)	16 609 (0-40 062)	46 525 (8567-84 482)	123 976 (68 926-179 026)
**2012**	8547 (0-20 803)	10 099 (0-23 279)	23 692 (0-50 685)	23 923 (0-58 111)	36 011 (0-78 181)	100 537 (37 294-163780)
**2013**	10 229 (0-20 683)	9558 (0-20 919)	39 529 (13 705-65 354)	16 037 (0-40 301)	53 926 (11 940-95 912)	126 282 (69 213-183 351)
**Average (2010-13)**	11 203 (0-24 998)	11 025 (0-24 500)	34 275 (6178-62 371)	22 395 (0-53 576)	48 194 (5283-91 105)	127 092 (64 046-190 139)

CI – confidence interval

## DISCUSSION

Our findings demonstrate that influenza virus activity is associated with respiratory and circulatory deaths in India. The rate of inﬂuenza-associated death was highest among those aged ≥65 years followed by children <5 years. Inﬂuenza-associated mortality estimates which were derived using national data sets on mortality and influenza surveillance consistently showed more deaths associated with influenza A viruses across the years.

Prior to analysing our data, we reviewed all available data sources in India and possible methods for our analyses to determine the best approach for the analyses [8]. We determined that generalized linear models using influenza viral surveillance data would provide the most reliable estimates of influenza-associated mortality in India. Our findings are consistent with observations from other countries, such as China[11], Hong Kong[22],Thailand[12], Bangladesh[13], South Africa,[23] and the Americas[10] (Table S4 in the **Online Supplementary Document**). While our point estimates of all age influenza-associated circulatory deaths (6/100 000, 95% CI = 1-10) were higher than those from previous studies in Thailand [12] (0.8/100 000, 95% CI = -15-16) and Hong Kong [22] (2/100 000, 95% CI = 0.6-4), the estimates were within the confidence intervals of other studies, indicating similarity with previously published estimates. One possible factor contributing to the variation across these studies is that these were conducted using data from different years (Thailand: 2006-11; Hong Kong: 1998-2009 and India: 2010-13). Other factors that may contribute to differences in mortality impact between countries include access to medical care and antiviral drugs, inﬂuenza vaccination coverage, and co-circulating bacterial and viral pathogens [24].

In addition to comparing our findings to other countries, we compared the mortality patterns by age group to hospitalization patterns by age group in India. We observed higher inﬂuenza-associated death rates among those aged ≥65 years followed by children <5 years, which correlates with earlier reported influenza-associated hospitalization rates in India. The Hirve et al. study found higher hospitalization rates in adults aged >60 years, followed by those aged 1-4 years [4]. While these burden estimates are comparable, the Hirve et al. study was not nationally representative and future efforts should be considered to calculate national estimates of influenza-associated hospitalization.

A strength of this study is that we were able to estimate influenza-associated respiratory deaths for children <5 years, which is not possible in many settings because there are few deaths by week or month in this age group for time series modeling. We compared our estimates for children <5 years (7.4-12.5/100 000 population) with South Africa [25] (6-13/100 000 population) and Bangladesh [13] (6-13/100 000 population) and found that these rates were similar. Other comparisons for children <5 years across LMIC settings were not possible. Mortality rates for children <5 years are important for developing country settings since child death rates are higher and vaccination programmes primarily focus on this age group [26]. Earlier estimates reported that 99% of global influenza-associated deaths in children <5 years occurred in developing countries [1,9]. Our Indian estimates contributed to a recent global estimate of 9243-105 690 annual influenza-associated deaths for this age group, which was estimated for 92 countries with high child respiratory infection mortality [1].

Although, there were peaks in inﬂuenza-associated mortality in India during 2010 through 2013, in distinction to temperate climates, the timings were inconsistent. This reiterates that year-round circulation of inﬂuenza viruses occurs in India as reported by earlier studies [2], and this circulation pattern prevents us from defining definitive seasonal mortality or epidemic patterns. The presence of multiple peaks in influenza virus seasonality indicates the importance of incorporating viral surveillance data into statistical models (a strength of our methods) to avoid overestimating the burden of influenza outside of the typical seasonal activity. Another strength of the model was providing mortality estimates by virus types and subtypes. One limitation of our study was that virological data were from only 10 sentinel centers across India and may inadequately represent the variation in influenza circulation across the country. In addition, we were unable to use age-specific influenza percent positive in models to estimate influenza mortality. This may have resulted in an overestimate or underestimate of influenza mortality in some age groups. In the future, if the network for influenza surveillance is expanded further, more representative patterns could help better define seasonality for influenza virus circulation and could provide enough specimens collected and tested by age group to use age-specific percent positive in analysis. An expanded system could also help to determine the need to further stratify mortality analyses by region within the country to account for different circulating patterns of influenza viruses. While we did have sufficient viral surveillance data by influenza subtype to estimate the burden of influenza virus subtypes, we were unable to differentiate the burden due to the different influenza lineages. In our estimates, influenza B accounted for about a quarter of respiratory and circulatory deaths in those aged <65 years. Thus, additional viral surveillance testing for B lineages along with research to understand the burden of influenza B, especially in younger age groups, could help to refine future influenza-associated burden estimates [27].

Additionally, peak respiratory and circulatory mortality occurred during winter months, which was outside of the typical influenza season and may potentially be explained by respiratory syncytial virus (RSV) circulation or air pollution due to temperature inversion in colder months [28]. However, increased circulatory mortality may occur during and outside of the typical influenza season. The increase in circulatory mortality may be due to cold temperature during winter months potentially leading to increased blood pressure and thrombus formation [29]. Other respiratory bacterial or viral pathogens such as S. pneumoniae and parainfluenza may also contribute to these deaths [24,30]. Some recent global estimates have shown the sizeable amount of mortality due to RSV [31] and air pollution [32]. These variables were not included in our models due to absence of RSV and other respiratory pathogens surveillance data or accurate temperature, humidity and air quality data at the national level. Future models would benefit from inclusion of data on these variables if data become available to provide more accurate estimates of influenza-associated death. Further, using national level week averages for air quality measures, temperature and humidity may not be as useful as using these variables for more defined geographic regions for these analyses and including measures of air quality, temperature and humidity on a smaller scale. Despite these limitations, our analysis indicated that few deaths were associated with influenza outside of the typical influenza season, which helps to demonstrate that our results do not misattribute deaths to influenza outside of the typical circulation period and reinforce the importance of including viral surveillance data to capture influenza virus circulation patterns in estimation models.

Lastly, the mortality data are from a nationally representative survey instead of the routine death reporting and certification system representing the entire population of India. Data sources and methods utilized for this analysis were chosen based on a detailed evaluation of possible data sources available in India for estimation of influenza-associated mortality conducted earlier by the study authors with a comparative analysis of the strengths and limitations of the data sources and various methods for influenza mortality estimation [8]. A limitation of the SRS data was that only four years of data were available for this analysis at the time of our data request. Though our models were evaluated to ensure that four years of mortality data were sufficient for our analyses and were found to be reliable, additional years of mortality data could improve models and allow for more stability and robustness of both the model and the estimates produced. A potential benefit of using death data from a large representative survey implementing verbal autopsy to model influenza-associated deaths is the possible application in other countries with limited mortality data or without comprehensive vital records registration systems. However, findings should be interpreted with the understanding that death data collected using verbal autopsy may be limited by the high proportion of ill-deﬁned causes of death in older adults [33] thereby suggesting that our estimates in adults aged ≥65 years (63.5% of total deaths) may be an underestimate. National-based vital records systems accounting for the entire population are needed to better estimate the burden of influenza on death in India. Also, because India is a geographically large and diverse country, further analyses by region could provide better information for the possible variations in presence of influenza and subtype by regions.

## CONCLUSIONS

We observed a high burden of influenza-associated mortality among adults ≥65 years and children <5 years. This study represents an important contribution to quantifying the death burden posed by influenza viruses in India, which may be useful to advocate for strategies for prevention and control, including the use of vaccines. This also identifies older adults and under-5 children as a high burden group to be considered for vaccination in the future vaccine policy recommendation of the MoHFW. Improvements to influenza virus surveillance and vital registration systems in India would increase the robustness of future estimates of influenza-associated disease burden.

## Additional material

Online Supplementary Document
