# Supplementary material for: Burden of influenza-associated respiratory and circulatory mortality in India, 2010-2013
Source: J Glob Health. 2020 Apr 15;10(1):010402. doi: 10.7189/jogh.10.010402 (PMC7182391; doi:10.7189/jogh.10.010402)
Supplement: Online Supplementary Document [file jogh-10-010402-s001.pdf]

Supplementary Material

Table S1: Description of the model fitting procedure

Serial. No.	Model description	AIC (Akaike Information Criterion)			Statistically Significant terms (p<0.05)		
		<5 years	5-64 years	≥65 years	<5 years	5-64 years	≥65 years
1.	*Final model (log link with influenza percent positive and no lag)*	1295	1313	1527	Five terms significant [intercept, time, sine, cosine, A(H3N2)]	Two terms significant (intercept & cosine)	Four terms significant [intercept, cosine, A(H3N2), A(H1N1 pdm09)]
2.	*Spline* (*in final model, splines used instead of harmonic terms)	1303	1303	1519	8 splines: 6 significant<br><br>intercept: significant<br>A(H3N2): significant	19 splines: 8 significant<br><br>intercept: significant	12 splines: 8 significant<br><br>intercept: significant<br>A(H3N2): significant<br>A(H1N1pdm09): significant
3.	Linear link** (in final model, log link replaced with linear link)	Model didn't converge	1316	1537	Model didn't converge	2 significant (intercept & cosine)	3 significant [Cosine, A(H3N2), A(H1N1 pdm09)]
4.	stdPP*** (In final model, standardized percent positive used instead of percent positive)	1300	1314	1531	4 significant (intercept, time, sine, cosine)	3 significant (intercept, time, cosine)	4 significant [intercept, cosine, A(H3N2) & A(H1N1pdm09)]
5.	Lag 1 (In final model, viral data was lagged by one week)	1291	1310	1516	4 significant (intercept, time, sine, cosine)	3 significant (intercept, time, cosine)	4 significant [intercept, cosine, A(H3N2), A(H1N1pdm09)]
6.	Lag 2 (In final model, viral data was lagged by two weeks)	1287	1303	1504	4 significant (intercept, time, sine, cosine)	3 significant (intercept, time, cosine)	3 significant [intercept, cosine, A(H3N2)]

* Spline: allows for additional seasonal variations as compared to the cyclical pattern use of harmonic terms (sine and cosine). Thus: (Example: Muscatello 2013 (1))
**Linear link assumes an additive relationship between the exposure to influenza and resulting mortality. (Example: Tempia 2015 (2))
*** Unlike percent positive, which was calculated by dividing the total number of influenza positive specimens for month X by the total number of specimens collected for month X, standardized percent positive was calculated by dividing the total number of influenza positive specimens for month X by the total number of specimens collected for the entire year. The standardized percent positive is a way to account for the variability in the number of specimens processed/tested/collected for a given week during the influenza season and for low number of sample collection.

Table S2: Annual number of deaths reported through SRS for India 2010-2013 (Source: SRS)

	Total Deaths (all cause)	Respiratory Deaths (%) (J00-J99)				Circulatory Deaths (%) (I00-I99)		
Year		Total*	<5	5 to 64	≥65	Total*	<65	≥65
2010	44705	5151 (11·5)	1099 (21·3)	1199 (23·3)	2853 (55·4)	9410 (21·0)	4444 (47·2)	4966 (52·8)
2011	44780	5195 (11·6)	956 (18·4)	1231 (23·7)	3008 (57·9)	10030 (22·4)	4794 (47·8)	5236 (52·2)
2012	45573	5115 (11·2)	870 (17·0)	1239 (24·2)	3006 (58·8)	10905 (23·9)	5240 (48·1)	5665 (51·9)
2013	44128	4972 (11·3)	749 (15·1)	1121 (22·5)	3102 (62·4)	10540 (23·9)	4790 (45·4)	5750 (54·6)
Total	179186	20433 (11·4)	3674 (18·0)	4790 (23·4)	11969 (58·6)	40885 (22·8)	19268 (47·1)	21617 (52·9)

*% of total deaths (all cause)

Table S3: Annual sum of total specimens tested and specimens positive for influenza by subtypes in India, 2010-13 (Source: ICMR-NIV Influenza surveillance network)

	Total specimens tested	Influenza positive (%)	Influenza A(H3N2) (%)	Influenza A(H1N1pdm09) (%)	Influenza B (%)
2010	6660	859 (12·9)	51 (5·9)	408 (47·5)	399 (46·4)
2011	8155	1181 (14·5)	661 (56·0)	141 (11·9)	379 (32·1)
2012	13240	1596 (12.1)	137 (8.6)	520 (32.6)	939 (58.8)
2013	9761	1135 (11.6)	690 (60.8)	258 (22.7)	182 (16.0)
Total	37816	4771 (12.6)	1539 (32.3)	1327 (27.8)	1899 (39.8)

*% of total specimens tested

Table S4: Comparison of estimates with studies from other countries

Country	Study Duration	Method	Age	Excess deaths/100,000/year (95%CI)		
				Respiratory deaths	Circulatory deaths	Respiratory & Circulatory
India	2010-13	Negative binomial	all age	4·7 (1·9-7·5)	5·8 (1·5-10·2)	10·5 (5·3-15·7)
			≥65	51·1 (9·2-93·0)	71·8 (7·9-135·8)	122·9 (46·5-199·4)
			<5	9·8 (-2·3-21·8)<br>Range (7·4-12·5)		
Southern China (3)	2010-2012	Negative binomial	all age			11·4 (9·4-13·4)
			≥65			146·9 (120·7-173·0)
Thailand (4)	2006-2011	Negative binomial	all age	4·3 (-10·1-19·0)	0·8 (-14·7-16·0)	
			≥65	42 (-93-178)	11 (-120-138)	
Bangladesh (5)	2010-12	mortality multiplier	All age	Range: 6-11		
			>60	41-88		
			<5	6-13		
Hong Kong (6)	1998-2009	Linear regression	all age	5·8 (4·1-7·3)	2·0 (0·6-3·6)	
			≥65	49·6 (37·1-61·0)	18·9 (9·6-31·2)	
South Africa (2,7)	1998-2009	Linear regression	5-64	8·5 (5·8–11·2)	7·5 (5·8–12·3)	
			65-74	43·4 (28·9–59·1)	52·1 (37·3–66·4)	
			≥75	132·3 (92·9–174·2)	167·4 (128·2–199·2)	
			<5	Range: 6–13		
Americas (8)	2002-2008	Serfling and mortality multiplier	All age			9·6 (4·6-18·1)
			65-74			31·9 (13·0-65·7)
			≥75			161·8 (87·2-288·3)

References (Supplement):

1. Muscatello DJ, Newall AT, Dwyer DE, MacIntyre CR. Mortality Attributable to Seasonal and Pandemic Influenza, Australia, 2003 to 2009, Using a Novel Time Series Smoothing Approach. PLoS One. 2013;8(6):e64734.
2. Tempia S, Walaza S, Viboud C, Cohen AL, Madhi SA, Venter M, et al. Deaths Associated with Respiratory Syncytial and Influenza Viruses among Persons ≥5 Years of Age in HIV-Prevalent Area, South Africa, 1998–2009 1. Emerg Infect Dis. 2015;21(4):600–8.
3. Wang H, Fu C, Li K, Lu J, Chen Y, Lu E, et al. Influenza associated mortality in Southern China, 2010-2012. Vaccine. 2014;32(8):973–8.
4. Aungkulanon S, Cheng P-Y, Kusreesakul K, Bundhamcharoen K, Chittaganpitch M, Margaret M, et al. Influenza-associated mortality in Thailand, 2006–2011. Influenza Other Respi Viruses. 2015;9(6):298–304.
5. Ahmed M, Aleem MA, Roguski K, Abedin J, Islam A, Alam KF, et al. Estimates of seasonal influenza-associated mortality in Bangladesh, 2010-2012. Influenza Other Respi Viruses. 2018;12(1):65–71.
6. Wu P, Goldstein E, Ho LM, Yang L, Nishiura H, Wu JT, et al. Excess Mortality Associated With Influenza A and B Virus in Hong Kong, 1998–2009. J Infect Dis. 2012;206(12):1862–71.
7. Tempia S, Walaza S, Viboud C, Cohen AL, Madhi SA, Venter M, et al. Mortality associated with seasonal and pandemic influenza and respiratory syncytial virus among children <5 years of age in a high HIV prevalence setting - South Africa, 1998-2009. Clin Infect Dis. 2014;58(9):1241–9.
8. Cheng P-Y, Palekar R, Azziz-Baumgartner E, Iuliano D, Alencar AP, Bresee J, et al. Burden of influenza-associated deaths in the Americas, 2002–2008. Influenza Other Respi Viruses. 2015;9(Suppl 1):13–21.
